# Functional effects of the spatial-varying lens mechanical properties in accommodation

**DOI:** 10.1088/2515-7647/ad3e55

**Published:** 2024-07-02

**Authors:** Justin Schumacher, Raymundo Rodriguez Lopez, Kirill Larin, Fabrice Manns, Giuliano Scarcelli

**Affiliations:** 1 Fischell Department of Bioengineering, University of Maryland, College Park, United States of America; 2 Department of Biomedical Engineering, University of Houston, Houston, TX, United States of America; 3 Department of Biomedical Engineering, University of Miami, Coral Gables, FL, United States of America; 4 Ophthalmic Biophysics Center, Bascom Palmer Eye Institute, University of Miami Miller School of Medicine, Miami, FL, United States of America

**Keywords:** lens biomechanics, presbyopia, ophthalmology

## Abstract

Lens biomechanical properties are critical for our eyes to accommodate. While it is well understood that lens mechanical properties change with age, different experimental techniques have been used over the years, with varying results on how the lens modulus changes. In this study, we developed a spatial-varying elasticity model to characterize the overall elastic modulus of the lens and establish its effect on accommodation. First, to validate the model, ex vivo porcine lenses underwent compression testing using biopsy punches of different diameters to change the percentage of nucleus within samples. Importantly, we found that, indeed, changing nucleus/cortex spatial ratio produces dramatic (∼7-fold) increase in overall sample modulus. Comparing the model with human lens spatial ratios, we demonstrate how changing spatial mechanics are more influential than peak modulus changes on overall elastic modulus. Next, in vivo clinical measurements of the spatial-varying lens modulus were used to generate a simplified mechanical-optical model of accommodation. We defined an ellipsoid lens with patient-derived modulus and geometry measurements, and a statics simulation and ray tracing analysis were performed through the deformed and undeformed lens. The resulting accommodation estimates agree with general accommodation expectations.

## 1. Introduction

To react to near and far stimuli, healthy human vision requires the crystalline lens to make significant changes to both size and shape to adjust its focal length in a process known as accommodation. In presbyopia, this dynamic accommodation system fails, and affects everyone as they age [1]. Established evidence suggests that the stiffening of the lens is critical to the inability to accommodate [2–6]. However, despite this knowledge, current therapies do not treat the biomechanical nature of presbyopia and the major barrier to progress is in this direction is the lack of comprehensive characterization of the aging lens mechanics [7].

The human lens is circumscribed by a capsular bag as a basal lamina, with epithelial cells moving apically inward and laying down a hexagonal fiber mass [8]. The lens nucleus consists of fiber cells that have enucleated and contain packed fiber crystallin proteins as they move closer to the lens center. Studies of lens biomechanical properties have been widely performed, but while there is wide agreement on the lens stiffening with age, there is no consensus on how the lens modulus changes and by how much. There are many measurement methods for investigating human lens biomechanical properties. Previous work has relied on methods including lens spinning [9, 10], compression testing [3], indentation [4, 11], acoustic radiation force [12], and Brillouin microscopy [6, 13, 14]. Early work on measuring lens stiffness or mechanical modulus changes suggested that nuclear modulus was lower than the cortex, and that with age the lens nucleus modulus would reach or surpass cortex modulus [4, 9–11]. In other studies, nuclear modulus is always higher than the cortex, and the nucleus modulus further increases with age or remains constant [6, 12]. Brillouin confocal microscopy has been the only technique to measure spatially resolved human *in vivo* lens biomechanical properties. Brillouin microscopy measures the inelastic scattering between acoustic phonons and optical photons, and relates the frequency change of light to a materials longitudinal modulus [15]. However, Brillouin microscopy measures a high-frequency longitudinal modulus, which so far only empirically relates back to the quasi-static Young’s elastic modulus [5, 16]. As a result, most Brillouin microscopy work has reported relative changes in material mechanical properties. Interestingly, the Brillouin shift of the lens nucleus and cortex remains relatively constant with aging, with the nucleus being stiffer than the cortex at all ages, and the lens stiffening with age is dominated by a remarkable spatial variation in the Brillouin shift changes with age [6, 13, 14]. Despite this knowledge of *in vivo* lens spatial variation, no studies have validated or utilized the spatial variation in their understanding of lens overall biomechanical properties, which would impact the accommodation mechanism.

One important application of lens biomechanical measurements is the validation of the structure-function relationship between changes in mechanical properties and their effects on visual outcomes, namely accommodation responses. This is commonly done through finite-element methods (FEM) and mechanical modeling to support different theories of accommodation, such as Helmholtz [17], Coleman [18], and Schachar [19, 20]. While experimental studies provide overwhelming confirmation of the Helmholtz theory, many modeling approaches still rely on measurements taken from techniques that report the cortex as stiffer than the nucleus [21–25]. There are currently no approaches that take advantage of human *in vivo* measurements of stiffness for consideration of accommodation theories. Proper analysis of this structure-function relationship is important to understand presbyopia and therapeutic targets for novel intervention, and so effort is made to validate this relationship in this work. A validation of the spatial variations in lens mechanical properties on overall mechanical properties is needed to understand this structure-function relationship.

To date, the *in vivo* spatial-varying mechanical properties of the lens have not been utilized in understanding overall lens mechanical characterization, and *in vivo* data has not been applied to validate the structure-function relationship on accommodation. The purpose of this study is to measure and demonstrate the effects of the spatial-varying lens modulus on the overall lens elastic modulus. Porcine lenses share similarity with human lenses and their spatial-varying mechanical properties and will be used as a lens model for validating this measurement methodology. The paper is organized as follows: First we derived a relationship between a spatial-varying lens modulus and overall lens modulus using integrals of elasticity. Next, porcine lenses were measured with gold standard compression testing while removing outer cortex layers to demonstrate the increase in overall lens modulus as lower modulus layers are removed. In addition, an empirical relationship between the overall elastic modulus and averaged Brillouin modulus is shown, critically validating Brillouin microscopy’s sensitivity to spatial varying mechanics of the lens. Furthermore, the experimentally-validated analytical model of the spatial elastic modulus is applied to human lens mechanical measurements to demonstrate the effects of changing spatial variations and peak modulus changes on overall elastic modulus. Finally, we developed a structural-optical model of accommodation using clinical Brillouin datasets and overall lens modulus concepts developed earlier to validate the role of spatial mechanical properties and growing geometry on accommodation with aging.

## 2. Methods

### 2.1. Mechanical compression testing of crystalline lens biopsy punches

To validate the changes in overall elastic modulus with spatial-varying properties, we dissected *ex vivo* porcine crystalline lenses and performed uniaxial compression testing with various biopsy punches. Fresh eyes (∼6 months old, exact ages unknown) were acquired on the day of the experiment from a local butcher (Wagner Meats, Mt Airy, MD) and placed on ice until dissection began. Lenses were isolated through dissection the same day and were either measured directly or kept refrigerated and submerged in mineral oil until mechanical measurements could be taken within the next day. It has been previously observed that lenses kept on mineral oil retain their mechanical properties for up to 2 d [26]. Additionally, we have independently verified that measurements taken up to 24 h later using this protocol did not alter the overall mechanical measurements on whole lenses. On the day of mechanical measurements, the lenses were cleaned with PBS, wicked of excess solution, and either biopsy punched or left whole prior to dynamic mechanical analysis (DMA) compression testing (Q800 Dynamic Mechanical Analysis, TA Instruments). We measured lens samples with calipers for cylindrical diameter and thickness using the central axes of the sample prior to being placed on the DMA stage. From unbiopsied lens, the thickness was measured to be 5.95 ± 0.564 mm and the diameter was measured to be 9.72 ± 0.462 mm. The capsule of the lens was not maintained for this experiment as biopsy punches would destroy the capsule prior to testing. The DMA compression tests were conducted with a slow force ramp, with most samples using a maximum force ramp of 0.2 N min^−1^, and an initial static force of 0.001 N. Compression data collected with at least five points on the initial 20% strain were used for stress-strain analysis and measuring the Young’s modulus. Originally, 40 lenses were measured with two lenses not reporting valid compression data (2 or less datapoints on stress/strain curve) and were discarded. The remaining 38 lenses were collected across all conditions, and then 23 lenses were selected for the final analysis based on having at least five datapoints for analysis.

### 2.2. Validation of Brillouin measurements for lens elastic modulus estimation

To relate the overall elastic modulus to the Brillouin modulus, we measured Brillouin profiles of *in-situ* porcine eyes. Fresh eyes were acquired on the day of the experiment, and *in-situ* measurements were taken using an existing Brillouin confocal microscope [27]. Lenses were measured with successive axial scans 1 mm apart, and the resulting peak axial and equatorial profiles were represented as a second-order polynomial. The averaged Brillouin shift along the equatorial profile was defined using an integral average of the parabolic fit:

\begin{align*}{\nu _{\text{B}}}\left( r \right) = {p_1} - {p_2}*{r^{2/3}}\end{align*}

where ${p_1}$ and ${p_2}$ are the coefficients from the parabola fitting and $r$ is the radius of the lens biopsy punch sample. The averaged Brillouin modulus was then calculated from the averaged Brillouin shift using the following equation:

\begin{align*}M^{\prime} = \frac{{{\nu _{\text{B}}}^2{\lambda ^2}\rho }}{{4{n^2}}}\end{align*}

where $\lambda = 780{\text{ }}nm$ is the source wavelength, $\rho = 1.183{\text{ }}g/c{m^3}$ is the density, and $n = 1.4$ is the refractive index [5]. The resulting Brillouin modulus was plotted against the overall elastic modulus, and the log-log empirical relationship was established using the following fit:

\begin{equation*}\log M{^{^{\prime}}} = a^*\log E^{\prime} + b\end{equation*}

where $a$ and $b$ are coefficients of the empirical relationship.

### 2.3. Structural-optical lens model of accommodation

To demonstrate the changes in overall elastic modulus due to spatial-varying mechanical changes and its effects on the structure-function relationship of the lens, we developed an accommodation FEM model in COMSOL Multiphysics software (COMSOL Inc, Burlington, MA) based on a clinical dataset previously published [6]. We modeled the lens as an ellipsoid centered at the origin, and with displacement constraints along axisymmetric planes to limit decentering of the lens during deformation. The equation for the axisymmetric ellipsoid used is given below:

\begin{align*}z\left( r \right) = \pm \frac{T}{2}\sqrt {1 - \frac{{{r^2}}}{{{R^2}}}} \end{align*}

where $T$ is the lens thickness and $R$ is the equatorial radius [28]. For each lens, we determined the thickness from the clinical dataset and the equatorial radius from the following age-related function based on another human lens relationship [23]:

\begin{align*}R = 4.07 + 0.0084^*Age\end{align*}

where $R$ is the equatorial radius in mm, and $Age$ is reported in years. From the clinical dataset, we obtained the two-compartment elastic modulus profile using the percent nucleus or central lens stiffness fraction (CLSF) defined with the following equation:

\begin{align*}CLSF = \frac{{2.6 + 0.031\left( {age - 28} \right)}}{{3.5 + 0.032\left( {age - 20} \right)}}\end{align*}

where the numerator and denominator slopes represent the rate of change in the central plateau and whole lens from *in vivo* Brillouin measurements [6], and the numerator intercept has been adjusted to match lens measurements of a 29 year old [13]. The CLSF was assumed to be valid for each semiaxis of an interior ellipsoid to model a nucleus, and the lens nucleus and cortex component’s elastic modulus was set at 10.6 kPa and 1.4 kPa respectively based on measured data [12]. To simulate the accommodation amplitude, we solved a dis-accommodative static mechanics problem using the initial geometry as the accommodated lens state, which is a common technique used to study these systems [23, 25, 29]. First, the thickness values of all lenses were corrected to an accommodated state by adding a thickness correction value of 0.5123 mm based on observed thickness changes in the youngest lens [30]. This value was chosen from a linear fit of the thickness changes from reported ages below 80YO that responded to an accommodative stimulus of 8D, which resulted in ${{\Delta }}T = 0.7447 - 0.01162*Age$. Next, a uniform total force was applied in the uniaxial direction along the optical axis dimensions of the lens, simulating the lens forces experienced by the capsule. This force placement was used to mimic the effects of stretching the capsule radially along the equatorial axis, which would translate forces along the contact normal with the lens surface. A total force of 0.02 N was applied uniformly to each surface to compress the lens for all cases and was chosen empirically based on inspection of the youngest lens model’s deformed geometry to ensure appropriate deformation changes were occurring for that age. While difficult to compare, the force selection is in general agreement with other reported values for forces acting on the lens [31].

From the undeformed and deformed geometry, the focal plane of the lens model was assessed using a ray-tracing simulation. A 50-element ray bundle parallel to the optical axis was sourced 10 mm before the lens geometry and directed along the optical axis. The resulting rays were traced through aqueous media with a refractive index of ${n_{{\text{aq}}}} = 1.336$, a lens refractive index of ${n_{{\text{lens}}}} = 1.42$, and an effective aperture was set at 80% of the lens diameter. The resulting ray positions after the lens were used to minimize the ray bundle’s root-mean-square (RMS) radial positions along the optical axis to identify the focal plane location. From the two focal lengths before and after deformation, the simulated accommodative amplitude in diopters ($AAD$) was determined from the following equation:

\begin{align*}AAD = \frac{{1.336}}{{{f_{{\text{unstretched}}}}}} - \frac{{1.336}}{{{f_{{\text{stretched}}}}}}\end{align*}

using the difference in diopters from the two ray bundle focal planes.

## 3. Results

### 3.1. Spatial-varying model of elasticity for crystalline lens

**Figure 1.**
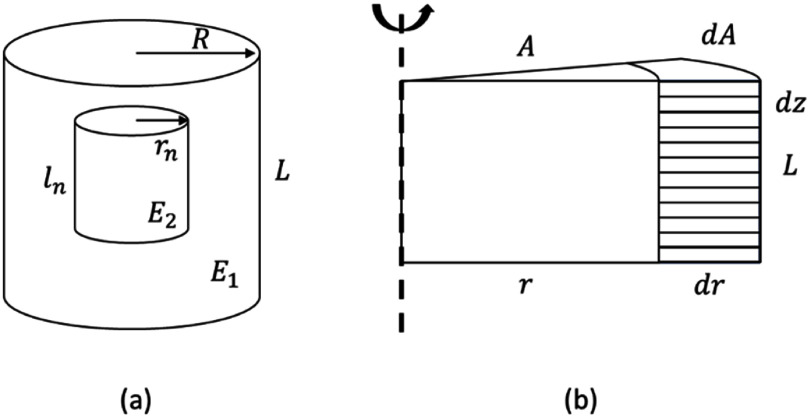
Diagram of a two-component cylindrical sample (a) under uniaxial compression and (b) its axisymmetric model.

The overall effective elastic modulus of a spatial-varying material can be complicated to relate back to nucleus and cortex mechanics, as shown for the crystalline lens. To better characterize the mechanical properties of the lens, the overall elastic modulus was determined from uniaxial compression testing and related to a two-compartment elastic model using an integral definition of elasticity. From structural mechanics, the elastic spring constant $\left( k \right)$ can be related to the Young’s modulus $\left( E \right)$ using the following equation:

\begin{equation*}k = \frac{{EA}}{L}\end{equation*}

where $A$ is the cross-sectional area of the material under stress, and $L$ is the length of the material under stress. Now, if we consider a cylinder of radius $R$ and thickness $L$ under compression as shown in figure 1, and apply integral operations to the spring constants as a function of radius ($r$) and thickness ($z$) dimensions, we get the following relationships:

\begin{equation*}k^{\prime} = {\mathop \int \nolimits }{}k{\text{d}}r = \frac{{2\pi }}{L}\int\limits_0^R E\left( r \right)r{\text{d}}r\end{equation*}

\begin{equation*}\frac{1}{{k{^{^{\prime}}}}} = {\mathop \int \nolimits }{}\frac{1}{k}{\text{d}}z = \int\limits_{ - L/2}^{L/2} \frac{{{\text{d}}z}}{{EA}}\end{equation*}

where the spring constants can be solved for spatial variations in $E\left( {r,z} \right)$ along the cylinder. This formalism is analogous to the mechanical circuit model of compression, treating the radial dimension as springs in parallel and thickness dimension as springs in series.

Using the integral derivations above, we defined the crystalline lens as a two-compartment material consisting of cortex and nucleus, using the following piecewise function:

\begin{align*}E\left( {r,z} \right) = \left\{ \begin{array}{*{20}{l}} {{E_2},{\text{ }}\,\,\,\,r < {r_n},{\text{ }}z < \frac{{{l_n}}}{2}} \\ {{E_1},\,\,\,\,{\text{ }}else} \end{array}\right.\end{align*}

where ${E_2}$ and ${E_1}$ represent the nucleus and cortex modulus respectively, and ${r_n}$ and ${l_n}$ represent the radius and thickness of the nuclear compartment. Of interest to lens mechanical measurements is the ratio of nucleus and cortex stiffness, and so a gain factor ($g$) is introduced to relate the peak nucleus and cortex stiffness ratios:

\begin{equation*}{E_2} = g^*{E_1}\end{equation*}

where $g &gt; 1$ would represent a stiffer nucleus than cortex, so that ${E_2} &gt; {E_1}$.

### 3.2. Validating the spatial-varying lens model of elasticity with gold-standard stress-strain test

Both the spatial-varying modulus and proposed analytical model can be experimentally-validated by spatially sampling different regions of the lens. While the whole crystalline lens is closer to an ellipsoid than cylinder, cylindrical sections of a lens can be measured in uniaxial compression testing using a biopsy punch of various diameters, and cylindrical geometry assumptions can be used for measuring the overall elastic modulus of that section of material. The following derivation solves the two-compartment solution for the crystalline lens biopsy punch along the equatorial diameter.

Returning to the cylindrical case, we can understand the biopsy punch as an operation limiting the maximum radius $R$ contributing to the overall elastic modulus, so we can derive an expression for the overall elastic modulus as a function of biopsy punch radius. From the math, it is apparent that the integrals have trivial solutions in the thickness dimension for $r &gt; {r_n}$, so we can first solve the thickness integral to get the following piecewise function:

\begin{align*}E\left( r \right) = \left\{ \begin{array}{*{20}{c}} {\frac{{{E_1}L}}{{L + {l_n}\left( {\frac{1}{g} - 1} \right)}},\,\,\,\,{\text{ }}r < {r_n}{\text{ }}} \\ \quad\,\,\,\,{{E_1},\,\,\,\,{\text{ }}else} \end{array}\right.\end{align*}

where $E\left( r \right)$ is reduced to only a radial dependence. Noticing that the individual terms in the equation are not dependent on any radial dimensions affected by a biopsy punch, we can rewrite this as:

\begin{align*}E\left( r \right) = \left\{ \begin{array}{*{20}{l}} {{E_n},\,\,\,\,{\text{ }}r < {r_n}} \\ {{E_c},\,\,\,\,{\text{ }}else} \end{array}\right.\end{align*}

where it is understood that

\begin{align*}{E_n} = \frac{{{E_1}L}}{{L + {l_n}\left( {\frac{1}{g} - 1} \right)}}\end{align*}

and

\begin{align*}{E_c} = {E_1}\end{align*}

which makes the derivation more intuitive for later experimental validation. Using this expression for $E\left( r \right)$, we can now solve the radial integral to get the following relationship for the overall elastic modulus $E^{\prime}$ of the lens as a function of biopsy punch radius $R$:

\begin{align*}E^{\prime}\left( R \right) = \left\{ \begin{array}{*{20}{c}} \qquad\quad\qquad\quad\,\,\,\,\,\,{{E_n},\,\,\,\,{\text{ }}r < {r_n}} \\ {\left( {{E_n} - {E_c}} \right)\frac{{r_n^2}}{{{R^2}}} + {E_c},\,\,\,\,{\text{ }}else} \end{array}\right.\end{align*}

where the first term can be interpreted as a biopsy punch of only nucleus-dominated modulus, and the second term can be interpreted as a modulus dependent on the proportion of cortex present in the periphery.

**Figure 2.**
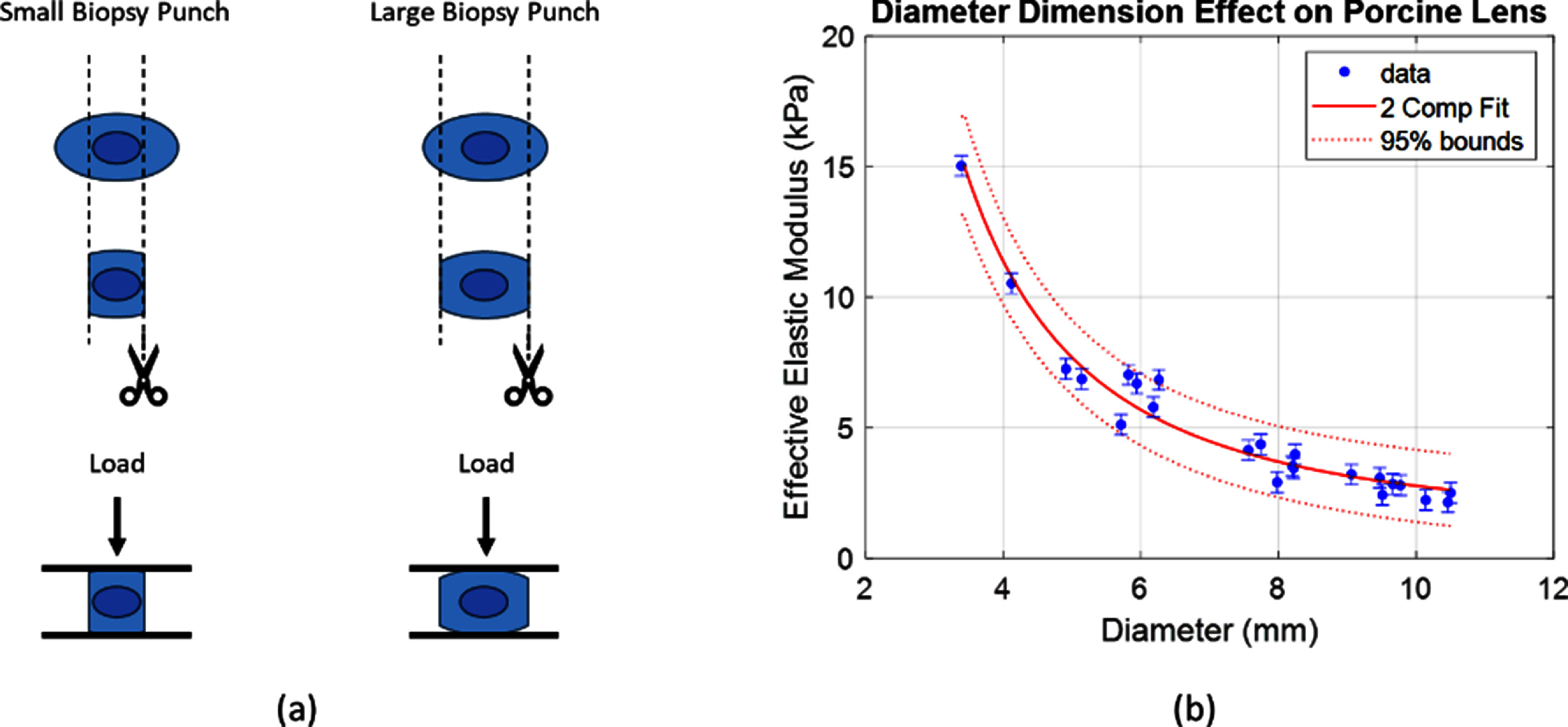
(a) Representative workflow for spatial-varying lens characterization. (b) Spatial-varying lens characterization with compression testing as a function of equatorial diameter and the resulting two-component lens model fit. Error bars are the standard deviation of measurements on the whole lens.

To measure spatial varying lens mechanical properties, lenses (*n* = 38) were biopsy punched and underwent mechanical testing as shown in figure 2(a). The resulting overall lens elastic modulus up to 20% strain was reported. The mean elastic modulus data ranged from 2.70 ± 0.392 kPa in the whole lenses, to 10.8 ± 2.52 kPa in the 4 mm biopsy punches. In addition, the variation in the smaller biopsy punched samples was larger. Even smaller biopsy punch data was observed to be less reliable due to visible buckling or slipping of the cylindrical samples.

From the data, we further analyzed lens biopsy punches as a function of the individual thickness and diameter parameters corresponding to the cylindrical cuts used. Lens samples from the DMA analysis with at least five datapoints contributing to the slope were used for analysis (*n* = 23). There was a clearly observed trend of increasing overall modulus with decreasing biopsy punch diameter, and overall elastic modulus values ranged from 2.14 kPa to 15.0 kPa as the biopsy punch diameter decreased. There was no observed trend with the thickness dimension of the biopsy punch, as expected. The data were fit to the elasticity equation to determine values of ${E_n}$, ${E_c}$, and ${r_n}$ that correspond to the porcine lens. The overall modulus results as a function of diameter and the fitting results are shown in figure 2(b). From the fitting, we found that ${E_n} = 15.0$ kPa, ${E_c} = 1.14$ kPa, and ${r_n} = 1.72$ mm. In addition, a more constrained fitting was performed using values of ${E_n} = 11.9{\text{ }}$ kPa and ${E_c} = 2.46$ kPa as reported in earlier work [5] to estimate the nuclear radius. The value of ${E_c}$ here was taken as an average of the reported anterior and posterior cortex values. The fitting results were less robust due to truncation of the extrema, but the value of the nuclear radius was still found to be ${r_n} = 1.76$ mm, in general agreement with the other nucleus radius fitting results.

### 3.3. Validation of Brillouin measurements for lens elastic modulus estimation

**Figure 3.**
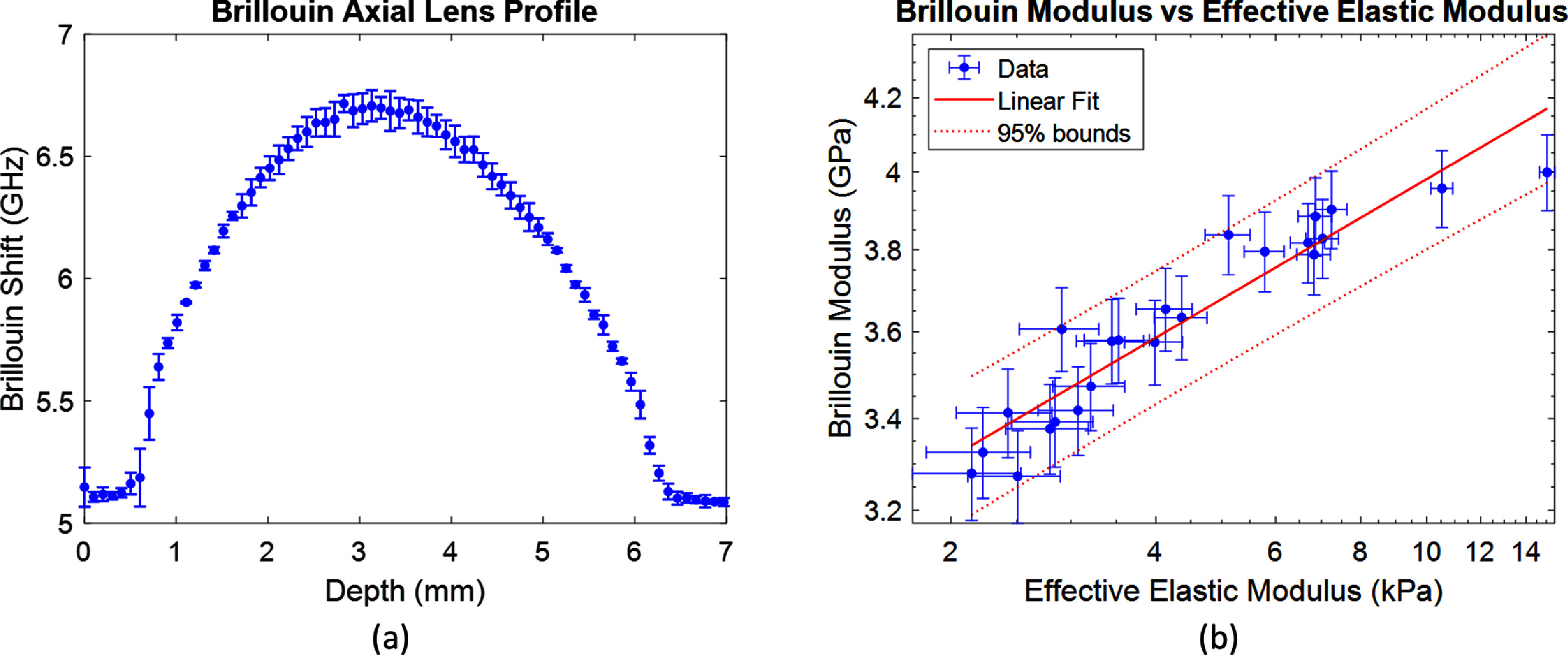
(a) Brillouin axial profiles from *in situ* porcine lens measurements. (b) Empirical log–log relationship between the average Brillouin modulus and overall elastic modulus. Error bars represent the sample standard deviation of measurements.

With the derivation and validation of the two-component mechanical model on overall lens elastic modulus completed, we moved towards understanding the relationship between the Brillouin modulus and overall elastic modulus. Intact porcine eyes and lenses were measured *in situ* with axial profiles spaced 1 mm apart to create a 2D map of Brillouin values. The resulting peak axial profiles for the three lenses are shown in figure 3(a). From the Brillouin measurements, we fit axial and radial profiles to a polynomial shape and used the values to generate an average Brillouin shift along an equivalent biopsy punch diameter. The resulting average Brillouin modulus as a function of the biopsy punch diameter was compared to the overall elastic modulus determined from mechanical testing, and the results are shown in figure 3(b). From the fit, the log-log calibration constants were found to be $a = 0.114$ and $b = 9.14$. From the data, there is a clear sensitivity of measured average Brillouin modulus to measured changes in spatial-variations encoded in the overall elastic modulus.

### 3.4. Validation of the changing spatial-varying modulus of elasticity

**Figure 4.**
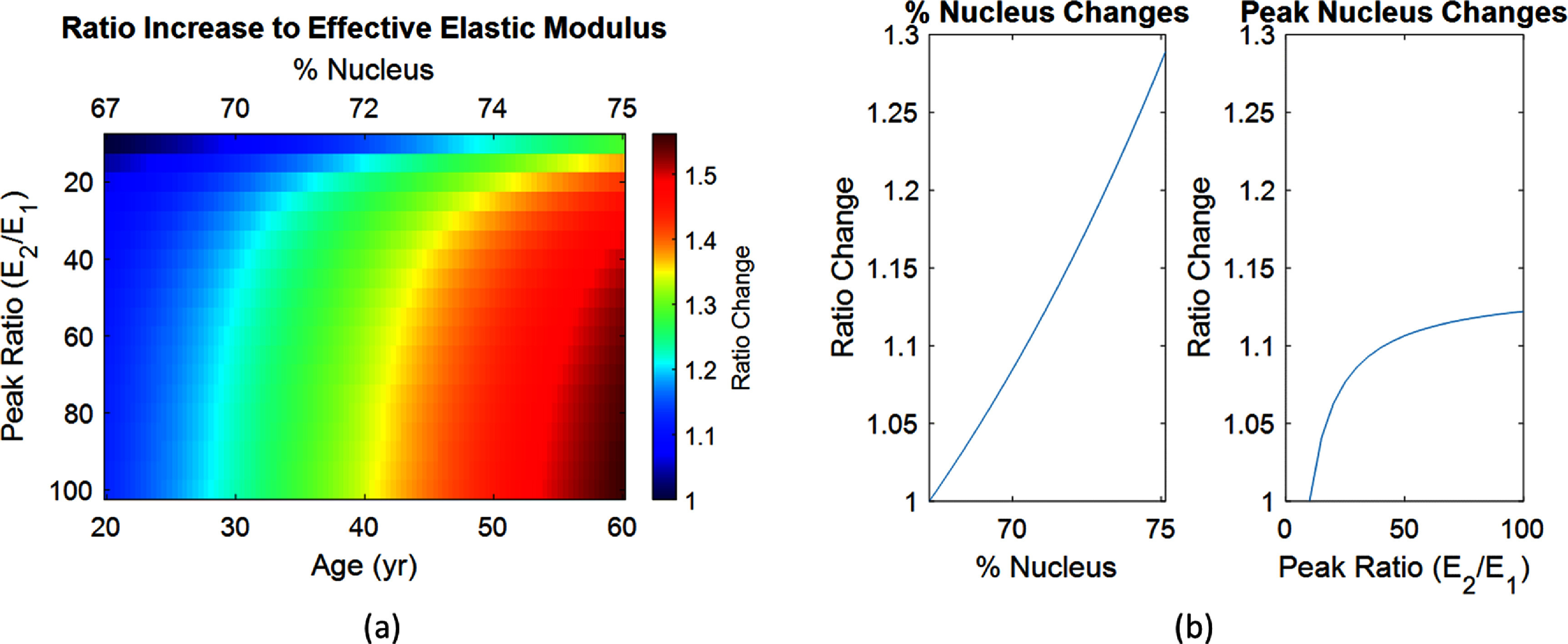
(a) Map of ratio increases to the effective elastic modulus as a function of both peak modulus nucleus/cortex ratio as well as the % nucleus. (b) Plot of ratio increases to the effective elastic modulus isolated to either changing the lens % nucleus or changing the peak modulus nucleus/cortex ratio.

With the understanding of both spatial-varying modulus and the reliability of Brillouin microscopy to assess the spatial mechanical properties of the crystalline lens, we explored the analytical mechanical effects of changing either the peak nucleus/cortex modulus ratio or the lens nucleus percentage. This is particularly relevant as we transition from a porcine model of a spatial-varying material to understanding factors that impact the human crystalline lens. An age range was assessed from 20YO to 60YO by defining an appropriate CLSF range, and the varying peak nucleus/cortex modulus value was assessed using a ratio of ${E_2}/{E_1}$ from 10 to 100. For each combination of age and peak ratio the effective elastic modulus was calculated. We show the resulting changes in figure 4(a). In addition, the isolated effects of exclusively changing either percent nucleus or peak ratio are compared in figure 4(b). From the analysis, there is a clear interpretation that changing nucleus percentage of the lens dominates the changes in effective elastic modulus.

### 3.5. Mechanical-optical lens model of accommodation

**Figure 5.**
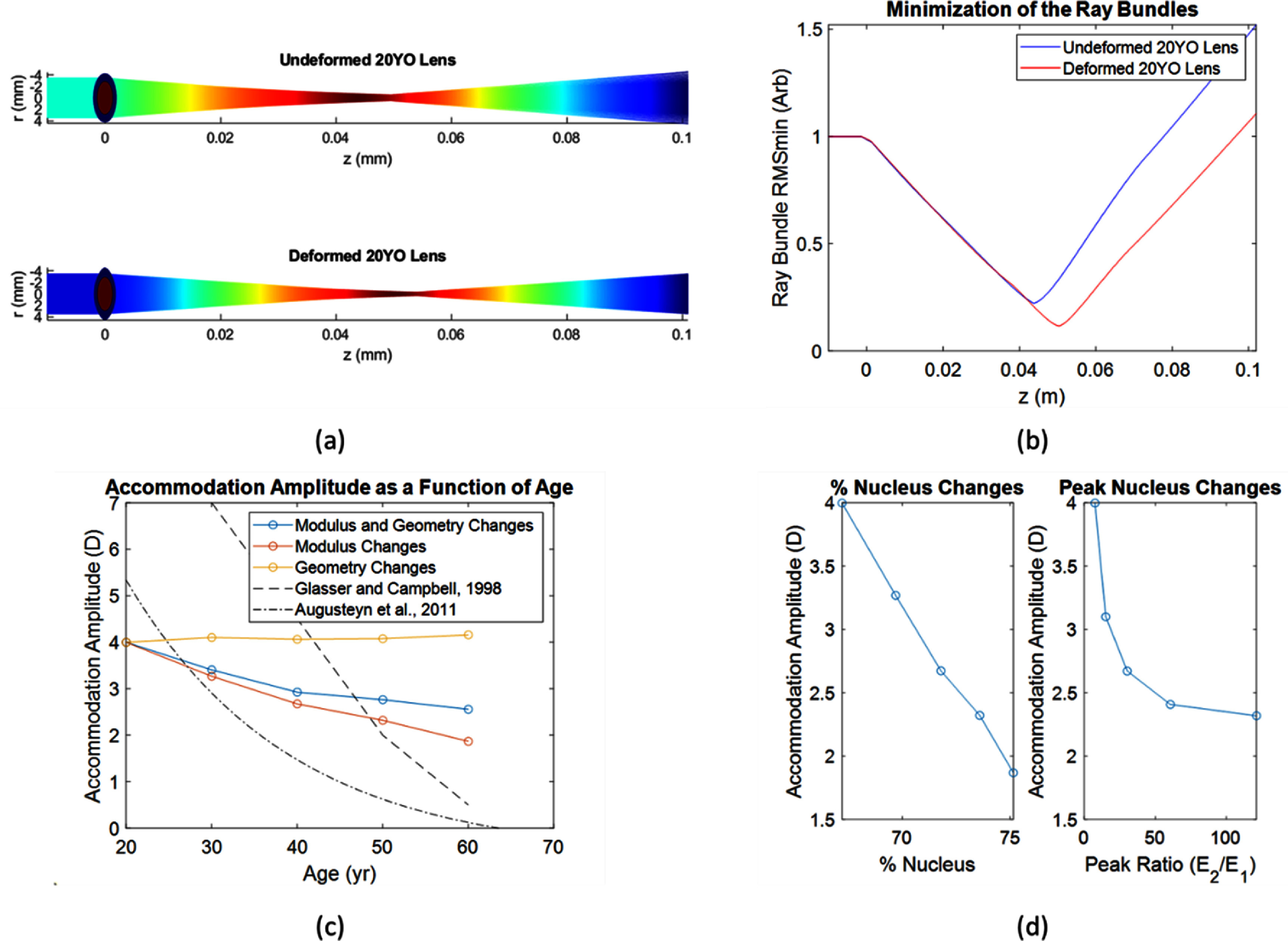
Structural-optical model of accommodation. (a) Representative workflow for a 20YO patient, showing the two-component modulus and changing focal planes with deformation. (b) The resulting RMS of the ray bundle was minimized, and the location corresponds to the focal plane of the lens. (c) The resulting accommodation amplitudes as a function of age. The model of accommodation was implemented with percent nucleus and geometry changes with age, percent nucleus changes alone, and geometry changes alone. The results are compared to reported human lens accommodation data on the same axis [2, 32, 33]. (d) Plot of accommodation loss due to either percent nucleus changes or peak nucleus/cortex ratio.

With experimental validation and analytical modeling of spatial mechanics applied to human lens changes with age, an important consideration remains the increase in lens geometry with aging, which may also contribute to accommodation loss. To validate the structure-function relationship of spatial-varying lens mechanics on functional lens accommodation in the aging lens, we constructed a structural-optical model based on human *in vivo* data of spatial-varying mechanical measurements. The optical axis data were taken from analysis of longitudinal modulus profiles of *in vivo* human lenses and are shown in table 1 along with an estimated thickness and equatorial diameter based on the equations above. We show the workflow for the 20YO subject in figure 5(a). The ellipsoid lens was first deformed using a structural mechanics model and compared to the undeformed geometry. There was a change in equatorial diameter of 0.61 mm, and a change in thickness of 0.23 mm. We next assessed the undeformed and deformed lens geometries with ray tracing focal plane simulations through the optical axis. We calculated the focal length using the RMS spot size along the optical axis, and the minimum RMS plane was identified and shown in figure 5(b). For the 20YO subject, the focal planes correspond to a change in 4.00 Diopters from undeformed to deformed.

**Table 1.** Clinical data used for the structural-optical model of accommodation. Age reported as years and dimensions as mm.

Age	Nucleus thickness	Lens thickness	CLSF	Lens diameter
20	2.35	3.5	0.672	8.48
30	2.66	3.82	0.697	8.64
40	2.97	4.14	0.718	8.81
50	3.28	4.46	0.736	8.98
60	3.59	4.78	0.752	9.15

From the dataset, we modeled aggregate patients from 20YO to 60YO to assess how the accommodation amplitude changed as a function of changing nucleus stiffness fraction, growing lens geometry, or both. The parameters used in the model are shown in table 1. We repeated the workflow shown in figures 5(a) and (b) for all patients and the accommodative amplitude was recorded for all subjects under each condition. The results are shown in figure 5(c). There was a clear drop in accommodation with age due to changing stiffness fraction, and stiffness fraction changes were necessary for agreement with human lens stretching trends. Geometry changes alone were unable to explain the accommodation changes with age in this modeling approach. Additionally, the comparison on percent nucleus changes and peak nucleus/cortex modulus ratio was repeated from figure 4(b) and is shown in figure 5(d). The results reinforce that spatial changes in the nucleus percentage may be equivalent in accommodation loss to large changes in the peak nucleus/cortex modulus ratio.

## 4. Discussion

Proper mechanical characterization of the lens remains an important consideration for understanding the role of spatial-varying mechanics. By combining both a two-compartment modeling approach to gold standard mechanical compression testing, we have explained the role of the individual spatial-varying mechanical moduli to the overall elastic modulus. Furthermore, this study is the first to use two-compartment models based on *in vivo* clinical measurements of the lens modulus to validate the structure-function relationship of the lens.

The porcine lens compression testing study was designed to show changes due to spatial variations in the lens, and since pigs are roughly the same age in our samples, another method was used to vary the modulus in a controlled manner. Biopsy punches have several advantages for this study. First, by removing the outside lens mass, the smaller biopsy punches result in larger nuclear percentages, which is expected based on observed in vivo clinical data [6]. In addition, the cylindrical geometry is conserved, which is convenient for compression testing as well as maintaining agreement with the two-compartment model cylindrical assumptions. This experimental configuration allowed for direct testing of varying overall elastic modulus due to spatial variation changes in a controlled manner, as porcine lenses measured were observed to be mechanically consistent with both Brillouin shifts as well as compression testing.

As seen in figure 2, the smaller biopsy punches trend towards higher overall elastic modulus. This effect is more clearly observed when the biopsy punch data is stratified by a measured biopsy diameter rather than grouped by punch diameter. There was no clear trend seen with the changes in lens thickness, as expected. The measurement and fitting results suggest that the biopsy punch diameters were not small enough to exclusively sample the nucleus, as we do not see a central plateau region. The main limitations in achieving this came from observed buckling failure with smaller cylindrical biopsy punches, which limited sampling of the overall elastic modulus when the equatorial dimension was 100% nucleus. Despite this physical limitation, the two-compartment model fitting was able to recover this plateau, and report both the nucleus and cortex elastic modulus. It is important to note that for a complete description of the lens model, the nucleus could not be solved with just diameter changes, but also thickness changes would need to be assessed. This was avoided here due to potential disruption of the tissue in the dimension of compression testing and remains an interesting topic for future work. It would be reasonable to hypothesize that the actual nuclear modulus is even higher than reported here, once the anterior and posterior cortex is removed from the lens sample. These derived elastic modulus values for nucleus and cortex are consistent with the lenses measured using Brillouin microscopy and optical coherence elastography [5], optical coherence elastography alone [34, 35], and equatorial stiffness trends are consistent with earlier porcine work by Erpelding *et al* using acoustic radiation force bubbles [36]. Porcine lens mechanical properties have previously been compared to middle-aged or older human lenses [36–38] in the literature based on their gradient properties, but overall properties are softer than human lenses [37]. The comparison of porcine lens characterization to human lens characterization was not directly assessed here, as this study serves as an experimental validation of this methodology for analyzing spatial-varying mechanical properties. Instead, the similarities in the porcine lens spatial-varying properties to human spatial varying properties allow for a good representative model of a spatial-varying material that can be assessed with elasticity integrals and biopsy-based compression testing.

The two-compartment model is based on equivalent spring systems in series and parallel, which serve as basic building blocks for many modeling techniques including FEM analysis for structural mechanics problems. Equivalent mechanical circuits were employed in this model as well, but instead of simplifying the problem into a few critical springs an integral form was used as shown in figure 1. This allows the model to adapt to various spatial-varying mechanical profiles by changing the dependence of $E\left( {r,z} \right)$. While two-compartment modeling was successfully validated in this study, experimental validation was only performed on radial biopsy punches. A critical assumption was made that the spatial-varying profiles of axial and radial springs behave identical in the lens. Since axial biopsy punch data was not acquired in this validation study to maintain tissue integrity along the axis of mechanical testing, the validation of the axial spatial-varying profile was not directly assessed. There may be a more accurate description of the axial spatial-varying modulus that remains a topic of future research.

With a validation of the two-component lens model, an empirical relationship between the overall elastic modulus and Brillouin modulus was achieved. Previous empirical relationships between the Young’s modulus and Brillouin modulus have been based on a log-log relation and have held with conditions that the material type measured is the same [5, 16, 39]. In spatial-varying materials, the average Brillouin shift was typically taken for comparison. In this work, the spatial-varying Brillouin profiles were fitted to a second-order polynomial before taken the integral average for comparison. Based on figure 3, there is a clear agreement with the empirical log–log relationship between the averaged Brillouin Modulus and overall elastic modulus, consistent with the other studies. The determined calibration constants reported in this work are $a = 0.114$ and $b = 9.14$, and are similar to the values reported for porcine lens tissue in other studies [5, 16]. There have been considerations in earlier studies that the Brillouin shift is not sensitive to changes in peak nuclear modulus with age. However, in consideration of the two-component model and the consistency of the porcine lens empirical calibration, it is likely that the averaged shift over the spatial-varying modulus is sensitive to overall elastic modulus changes. This makes sense for the lens since the integral definition of elasticity in the equatorial plane can be interpreted as a spatial average over that domain. For confocal Brillouin microscopy, this concept can be generalized to the following statement: the averaged Brillouin shift orthogonal to the optical axis is sensitive to changes in overall elastic modulus changes along the optical axis.

With experimental validation of the two-component model on the spatial-varying mechanical properties of the porcine lens, the analytical solution gives insight into the working principles of lens hardening mechanisms. One application of this analytical model is the sensitivity of changing a spatial percent nucleus or the peak nucleus modulus on overall elastic modulus of the lens. From figure 4, it is clear that changing from a percent nucleus of 67%–75% has a much larger effect on the overall elastic modulus than changing from 10:1 to 100:1 nucleus-to-cortex ratio peak modulus. This agrees conceptually with springs in series and parallel, as the central stiff spring asymptotically contributes to the overall spring constant in series. This estimate of the percent nucleus is important in this analysis, as larger nuclear percentages contribute to larger swings in the effective elastic modulus. While Brillouin confocal microscopy should be the most sensitive to this metric, clinical data is currently limited in our ability to properly estimate this percentage. Some studies have achieved this through structural imaging of the lens, commonly through optical methods [40–42]. Overall, the ability to properly estimate this percent nucleus *in vivo* remains an important next step in characterization of lens mechanical properties for human *in vivo* accommodation.

An important application of this work is validating the structure-function relationship of changing lens mechanical properties on the ability of the lens to accommodate under deformation. In addition, an aspect of changing lens geometry with age is not captured by the analytical modeling approach used in this study. The use of a structural-optical model allows for the comparison of spatial-varying two-component modeling, changes in peak nucleus/cortex ratios, and the effects of a growing lens geometry. The structural-optical model accommodation results are shown in figure 5 and offer insights into mechanisms of accommodation. The data suggest that accommodation loss is dominated by changes in spatial-varying elastic modulus, and that growing geometry changes with age do not play a large role in this modeling configuration. In addition, the accommodation values are comparable to previously measured human *ex-vivo* data [2, 33]. Due to the potential limitations of our approach by simplifying capsular action on the lens, we also verified geometry changes to the lens during compression. When comparing the dis-accommodative changes in lens thickness and diameter, there is a corresponding trend of decreasing thickness changes and diameter changes with age in all cases except for the growing geometry changes alone, where the magnitude of thickness change instead increases with age. This counterintuitive result is likely due to the increased axial geometry making the overall axial spring constant weaker. To compare the effects of the analytical model and structural-optical model, the study with changing percent nucleus and peak nucleus/cortex modulus ratio from figure 4(b) was repeated in figure 5(d) using accommodation loss instead of overall elastic modulus changes. The overall results are in general agreement, with the asymptotic curve present in both nucleus/cortex peak modulus plots in contrast to the changing spatial percent nucleus which remains linear. When comparing the magnitude of changes in lens thickness and diameter, the same trends are observed for the decrease in diameter changes being dominated by percent nucleus rather than peak nucleus/cortex ratios. However, the decrease in thickness changes were observed to be stronger in the peak nucleus/cortex ratios than percent nucleus. Thickness measurements remain a limitation of this approach, likely due to the optical axis of measurement directly experiencing the forces and axial effective spring constant. However, taken together, these data suggest that the spatial averaging in the radial dimension of the lens may be a more sensitive factor to accommodation loss.

There are several important aspects to consider in this modeling approach. One benefit to this workflow is that the entire lens geometry can be approximated by an ellipsoid. While the lens is typically represented with complicated shapes including polynomials and conics for modeling workflows [22–25, 29, 43, 44], this model only requires the equatorial diameter and thickness of the lens to define a shape. This may be a reasonable assumption as it has been shown that volume constraints alone with various lens geometries can all explain qualitative accommodation trends [28]. Not only are thickness parameters relatively easy to acquire in clinic, but the modeling computation step could be further simplified by using additional symmetry about the lens equatorial diameter. Another potential benefit of this approach is the use of raytracing instead of optical power equations. This increases the computational cost but allows for use of actual ray spot-diagram plots for analysis of focal planes. Future work into optical aberrations may also be explored in this type of analysis and would be of interest to researchers exploring novel therapies that alter shape or refractive index properties of the lens. When considering the collective results in the context of accommodation theories, the Helmholtz models are well supported by the overall elastic modulus changes explaining the loss of accommodation [17].

It is worth noting several assumptions and limitations with this simulation. First, the growing geometry changes alone are not affecting accommodation loss, which may be partially explained by the simplification of the lens to an ellipsoid. An ellipsoid has a characteristic sharpening of curvature in the periphery, and yet the mean curvature remains relatively constant. Because of this, it is likely insensitive to geometric changes to curvature. In addition, the clinical Brillouin dataset used measured lenses in a dis-accommodated state, and so the lens thickness may be biased compared with expected values. Indeed, our reported values after correction remain slightly higher than other studies for older patients [40, 45, 46], which may overstate the impact of growing geometry with age. Overall, the values for accommodation amplitude were lower than compared to equivalent lens stretching experiments [2, 32] and higher than other experiments [33, 47]. A better agreement of accommodative amplitudes to human data can be obtained by adjusting the lens modeling parameters for example to an ellipsoid geometry with lens refractive index of ${n_{{\text{lens}}}} = 1.376$ in air, which implies that simplified ellipsoid geometries are generally underpowered compared to experiments and may not completely account for optical effects. Thus, the ellipsoidal model used in this study is expected to have lower overall accommodation amplitudes. For detailed optical power and aberrations simulation, more modeling effort should be directed towards resembling the power in central lens shapes. An important assumption in the model was the simplification of zonule and capsular function. Previous work has demonstrated that the zonule modulus changes are small when considering effects on accommodation [48], and that ciliary contractile force is maintained with age [31]. While the capsule is known to significantly alter the overall mechanical properties of the lens [49], the capsule elastic modulus changes remain small with age compared to lenticular changes over presbyopia age ranges [50]. Because of these findings, rather than model zonule action on the capsule, our study directly assumes the action of the capsule in converting an equatorial stretching force into a force acting on the optical axis. An intuitive description for the force direction change can be explained using a tensioned membrane to apply a compressive load to an object beneath it [51]. Our model assumes this capsular force transfer, and instead the force for dis-accommodation is applied as a compressive force. This ciliary force value was assigned based on one example lens deformation and held uniform throughout every lens condition. When using the capsular force transfer, the goal of modeling should be carefully considered, as zonular function is not directly studied, and the capsular forces are applied directly where geometry measurements would be taken. In addition, we suggest caution in the interpretation of the magnitude of the capsular force transfer, as this is a fine-tuning parameter and the relation to true capsular forces was not compared in the study. When considering raytracing approaches, no aperture effects were considered from pupil changes with accommodation, as the lens was always assumed to work at 80% of the effective diameter. This may change the importance of lens center vs periphery optical power on spot-diagrams and focal plane selection. The lens was also measured with collimated light, which may not represent actual *in vivo* conditions where the incoming wavefront is partially converging due to corneal focusing. All these choices may lead to bias in the extent of geometry effects on accommodation, and likely contributed to differences with other lens stretching models. However, despite these shortcomings in the model, the geometry and two-compartment modulus changes can follow accommodation trends that were independently acquired. The refinements and development of this modeling approach is a topic of future work.

The present work represents an important step forward in characterizing the lens structure-function relationship, but there remain future improvements to our study. The current overall elastic modulus measurements are limited in the two-compartment model and was experimentally restricted to the equatorial diameter changes alone. In the future, careful lens dissections in both diameter and thickness dimensions can be performed to fit a complete model with respect to both geometry components. There is also a likely underestimation of the elastic modulus in the larger lens biopsy punches and whole lenses due to ellipsoidal geometry potentially dominating instead of cylindrical geometry. This can be partially mitigated by allowing flat plate contact before starting the compression test, but corrections may be better using Hertzian contact theory, and has been demonstrated by other researchers [52]. It was observed that for biopsy punches below 8 mm the stress–strain curves resembled shapes consistent with linear elastic models rather than Hertzian contact. In the structural-optical model of accommodation there was a limitation in the collection of data across multiple data sources. As a result, the present work had to collect geometry data, elastic modulus data, and accommodation data separately. In future studies, the collection of lens geometry and spatial-varying modulus data will be collected alongside clinical accommodation tasks for that patient. This will allow for a more direct patient-specific relationship between their unique lens characterization and functional effects on accommodation.

## 5. Conclusion

In conclusion, by considering the effects of spatial-varying lens mechanical properties, we demonstrated how changing spatial-variations in the lens lead to both structural and functional changes in the lens. For gold-standard compression testing, we showed how changes in percent nucleus along the lens equatorial diameter accounted for large changes in overall elastic modulus values for the porcine lens. In addition, these changes were used to validate a two-component model of lens mechanical properties, showing how overall lens mechanics can be explained by various combinations of high modulus nucleus and low modulus cortex components. Next, we used this validated model to empirically relate the overall elastic modulus and Brillouin modulus. Finally, we demonstrated through *in vivo* clinical datasets how changes in spatial-varying elastic modulus values and geometry lead to expected changes in accommodation trends. This ability to relate structural lens characteristics to functional accommodative outcomes is a powerful tool with applications in patient-specific monitoring and novel therapeutic planning for lens softening.

## Data Availability

All data that support the findings of this study are included within the article (and any supplementary files).
